# O-24 - PUBLIC HEALTH RESILIENCE FOR REMOTE-ISLAND MASS GATHERINGS: INSIGHTS FROM THE ORKNEY 2025 INTERNATIONAL ISLAND GAMES

**DOI:** 10.1093/pubmed/fdag046.021

**Published:** 2026-07-09

**Authors:** Mrs Kirsti Jones

**Affiliations:** NHS Orkney

## Abstract

**Background:**

Mass-gathering events present a recognised public health challenge, and international evidence highlights the need for enhanced surveillance, clear command structures robust risk assessment, and multisector preparedness. The 20th International Island Games Association (IIGA) island games event, held in Orkney in July 2025, was the largest multi-sport event ever hosted in the islands, bringing together over 2000 athletes, officials and visitors to the geographically remote and rural archipelago. For island settings, additional pressures such as limited surge capacity, transport constraints, and reliance on small health protections teams intensify these challenges.

**Objectives:**

To outline the public health planning, surveillance approach, and risk-mitigation measures developed for the IIGA Orkney 2025 Games, and to describe how international guidance was adapted for a remote and rural island context.

**Methods:**

Planning was undertaken through a multi-agency framework involving NHS Orkney's Health Protection Teams, Orkney Island Council, and the Island Games Organising Committee. Activities included strategic and rapid risk assessments, development of a dedicated HPZone context, outbreak-management planning, communication pathways and alignments with WHO mass gathering risk-assessment principles (WHO, 2015).

**Results:**

Key risks identified included gastrointestinal and respiratory outbreaks, the need for isolation capacity in shared accommodation, and staff resilience in small workforces. Mitigations included strengthened surveillance, pre-Games exercising, clear escalation pathways, and proactive information for visiting teams. Cruise-liner-related risks and the likelihood of high-consequence infectious disease importation were assessed as low. The work has also strengthened inter-agency coordination and preparedness systems, reflecting mass gathering legacy principles.

**Conclusions:**

Applying international mass gathering guidance within a remote island context highlights the importance of early planning, tailored risk assessment, and resilience-based approaches. The IIGA Orkney 2025 Games planning process demonstrates who small island systems can deliver safe mass events through collaboration, adaptability, and proportionate preparedness

References: WHO (2015) Public Health for Mass Gatherings: Key Considerations. Available at: https://cbrn-risk-mitigation.network.europa.eu/publications/who-public-health-mass-gatherings-key-considerations_en

